# Attention model of EEG signals based on reinforcement learning

**DOI:** 10.3389/fnhum.2024.1442398

**Published:** 2024-11-15

**Authors:** Wei Zhang, Xianlun Tang, Mengzhou Wang

**Affiliations:** ^1^School of Computer Science and Technology, Chongqing University of Posts and Telecommunications, Chongqing, China; ^2^School of General Education, Chongqing College of Traditional Chinese Medicine, Chongqing, China; ^3^Chongqing Key Laboratory of Complex Systems and Bionic Control, Chongqing University of Posts and Telecommunications, Chongqing, China

**Keywords:** reinforcement learning, strategy gradient, gradient descent optimization algorithm, gated recurrent units, EEG

## Abstract

**Background:**

Applying convolutional neural networks to a large number of EEG signal samples is computationally expensive because the computational complexity is linearly proportional to the number of dimensions of the EEG signal. We propose a new Gated Recurrent Unit (GRU) network model based on reinforcement learning, which considers the implementation of attention mechanisms in Electroencephalogram (EEG) signal processing scenarios as a reinforcement learning problem.

**Methods:**

The model can adaptively select target regions or position sequences from inputs and effectively extract information from EEG signals of different resolutions at multiple scales. Just as convolutional neural networks benefit from translation invariance, our proposed network also has a certain degree of translation invariance, making its computational complexity independent of the EEG signal dimension, thus maintaining a lower learning cost. Although the introduction of reinforcement learning makes the model non differentiable, we use policy gradient methods to achieve end-to-end learning of the model.

**Results:**

We evaluated our proposed model on publicly available EEG dataset (BCI Competition IV-2a). The proposed model outperforms the current state-of-the-art techniques in the BCI Competition IV- 2a dataset with an accuracy of 86.78 and 71.54% for the subject-dependent and subject-independent modes, respectively.

**Conclusion:**

In the field of EEG signal processing, attention models that combine reinforcement learning principles can focus on key features, automatically filter out noise and redundant data, and improve the accuracy of signal decoding.

## 1 Introduction

Significant progress has been made in the decoding and recognition of EEG signals based on deep learning network architecture. However, in order to achieve satisfactory accuracy, especially in cross subject EEG signal decoding tasks, the computational cost of model training is high, and it is still difficult to ensure satisfactory performance in decoding tasks (Liu et al., 2023; Song et al., 2023; Acharjee and Ahamed, 2024; He et al., 2023).

At present, attention mechanisms and transfer learning are widely applied in the decoding task of EEG signals, aiming to improve the accuracy of cross subject EEG signal decoding (Altaheri et al., 2023; Zhang et al., 2020; Liu et al., 2023). In neural networks, attention mechanisms autonomously learn a set of weight coefficients to emphasize regions of interest in the input and suppress irrelevant background regions. In this way, neural networks can focus more on key information related to the task, improving the performance and efficiency of the model (Vaswani et al., 2017). Reference (Altaheri et al., 2023) proposes an attention focused temporal convolution architecture aimed at optimizing the EEG data-driven motor imagery (MI) image classification task. This method integrates multiple strategies and significantly improves the performance of MI classification even while keeping the parameter size compact. This architecture utilizes a multi head self-attention mechanism aimed at accurately focusing and enhancing the most critical features in MI-EEG data. At the same time, a temporal convolutional network layer was introduced to deeply mine and extract complex temporal dimension advanced features. Zhang et al. (2020) utilized an attention mechanism based Long Short Term Memory (LSTM) neural network model, with deep learning and self -attention mechanisms at its core, to effectively capture and learn the complex information contained in EEG time series, thereby achieving precise differentiation and classification of left and right hand movement intentions. This approach not only broadens the perspective of EEG signal processing, but also provides new research ideas for brain computer interface technologies related to motor imagery (Zhang et al., 2020).

In recent years, there have been some advances in the research of attention mechanisms using neural networks for EEG signal processing, but there are still some challenges and limitations. For example, EEG signals usually have a low signal-to-noise ratio and are susceptible to various types of noise interference, such as motion artifacts, electromagnetic interference, etc., which increases the difficulty of signal processing. In addition, there are differences in brain structure and function among different individuals, resulting in high individual specificity of EEG signals, which makes it difficult to develop a universal EEG signal classification model. Meanwhile, EEG signals are highly complex and nonlinear, containing rich time-domain and frequency-domain features. How to effectively extract and utilize these features is a challenge.

In order to address these challenges in the field of EEG signal decoding, a new attention based task driven visual processing framework was developed using GRU neural network in this article. Our model considers the implementation of attention mechanisms in visual and EEG signal processing scenarios as a reinforcement learning problem, and is sufficiently versatile to be applied to static images, EEG signals, or as a perception module for agents interacting with environmental information such as static images and EEG signals.

This model is based on the GRU neural network, which first processes the sampling of various local features at different positions of the EEG signal to obtain local features, then integrates these local features to obtain global features, and finally forms the dynamic feature expression of EEG signal samples. That is to say, it is not processing the entire EEG signal sample at once, but at each step, the model selects the next location to process based on past information and task requirements. The number of parameters and the amount of computation performed in our model can be controlled independently of the size of the EEG signal, which is in stark contrast to convolutional networks. The time complexity of convolutional neural networks is linearly related to the dimension and number of channels of the EEG signal. We describe an end-to-end optimization process that allows for direct training of models for a given task and maximizes performance metrics that may depend on the entire decision sequence made by the model. This process uses backpropagation to train neural network components and policy gradients to address the non-differentiability brought about by the introduction of reinforcement learning.

Experiments have shown that our model can learn effective task specific strategies to determine its position on several image classification tasks and EEG signal classification problems. Our results also indicate that GRU neural networks based on reinforcement learning may be better than current mainstream baseline models in handling clutter and nonlinear non-stationary EEG signals.

## 2 Related work

Significant progress has been made in research on the attention mechanism of neural networks used for EEG signal processing in recent years (Xie et al., 2024; Liu et al., 2023; Hu et al., 2024; Cao et al., 2023). This attention mechanism enables neural networks to pay more attention to key information in the input sequence when processing EEG signals, thereby improving the accuracy and efficiency of the model. Firstly, in terms of EEG signal acquisition, the electroencephalograph collects EEG signals through electrodes and digitizes them for storage and analysis. However, due to the high level of noise and interference in EEG signals, it can have an impact on the performance and accuracy of neural network models. Therefore, denoising and filtering processing have become important preprocessing steps to remove unnecessary signals and noise. Furthermore, in terms of feature extraction of EEG signals, neural network models can automatically extract time and frequency features. These features include amplitude, frequency, phase, slope, and waveform, which help to understand the mechanisms and functions of brain activity.

After introducing attention mechanism into neural network models, the model can be more flexible and efficient in processing EEG signals. The attention mechanism allows the model to weight different parts of the input data, that is, assign different weights to different parts. In this way, the model can focus more on key information related to the task, while ignoring irrelevant or noisy information. For example, in EEG signal classification tasks, attention mechanisms can help models recognize features related to specific categories, thereby improving classification accuracy (Zhang et al., 2023; Han et al., 2023).

In addition, attention mechanisms can also be used for event correlation analysis of EEG signals. By simultaneously inputting stimulus or task time points and EEG signals into a neural network model, and introducing attention mechanisms, we can observe the changes in EEG signals before and after stimulus or task occurrence, as well as the model’s attention to different time points. This helps to study the brain’s response mechanisms to stimuli or tasks, and reveals the functional connectivity patterns of the brain network (Tang et al., 2024). In summary, research on the attention mechanism of neural networks used for EEG signal processing has made some progress, but still faces some challenges and limitations.

Future research can further explore how to optimize the structure and parameters of neural network models to improve their performance and accuracy in EEG signal processing tasks. Meanwhile, with the continuous development of deep learning technology, it is believed that more innovative methods will be applied in the field of EEG signal processing, providing strong support for the development of neuroscience and brain computer interface technology.

In recent years, reinforcement learning has been widely studied in EEG signal processing and analysis (Prabhakar and Lee, 2022; Mahapatra and Bhuyan, 2022). The strategy gradient method in reinforcement learning aims to optimize the policy function to enable agents to better adapt to the environment and achieve task objectives. An agent is an entity in reinforcement learning that can perceive environmental states, perform actions, and optimize its action choices based on environmental feedback (rewards or punishments). In EEG signal classification tasks, an agent can be seen as an intelligent system that predicts or classifies different brain activity states (such as attention, relaxation, thinking, etc.) by analyzing EEG signals.

The core of the strategy gradient method lies in directly modeling and optimizing the strategy to find the optimal strategy. The strategy here is a mapping that maps the state to the probability distribution of each action. The policy gradient theorem states that the gradient of the policy function is directly proportional to the product of the expected cumulative rewards based on this policy function, which provides a theoretical basis for optimizing strategies.

In the basic framework of reinforcement learning, agents take certain actions based on the state given by the environment and receive rewards based on the results of the actions. The goal of an intelligent agent is to learn a strategy that enables it to select the action that can receive the maximum reward in different states. The policy gradient method estimates the policy gradient and updates the policy parameters based on the gradient to gradually enable the agent to learn better policies.

In multi-agent systems, policy gradient methods can be extended to handle the learning and decision-making problems of multiple agents (Shin et al., 2024). The method based on multi-agent reinforcement learning can automatically extract and select key features from raw EEG signals, reducing reliance on manual experience each intelligent agent estimates policy gradients based on its own experience and updates policy parameters based on the estimated gradients. The interaction between intelligent agents is inevitable in this process, and through the strategy gradient method, intelligent agents can learn how to better act in interaction with other intelligent agents.

Overall, the machine learning framework using policy gradient methods provides an effective method for optimizing strategies in reinforcement learning, enabling agents to gradually learn the optimal behavioral strategy through trial and error.

The method proposed in this article is based on GRU continuously integrating signal features in the time dimension and making decisions on how to act, making the algorithm framework more universal. Meanwhile, the decision-making process adopts the strategy gradient method for end-to-end optimization, without relying on greedy action choices. We further demonstrate how this universal architecture can be used for efficient signal decoding in EEG signals. Introducing GRU (Gated Recurrent Unit) based on reinforcement learning to implement attention mechanism has the following advantages in solving the challenges faced by EEG signal processing:

1. The attention mechanism based on reinforcement learning can help models automatically focus on important features when processing EEG signals, ignoring noise and irrelevant information, thereby improving decoding quality. Reinforcement learning can adapt to different noise environments by continuously optimizing model parameters, further improving the robustness of the model.
2. Based on reinforcement learning attention mechanism, the model can be continuously optimized through interaction with the environment, learning the specific features of different individual EEG signals, which can enable the model to better adapt to individual differences.
3. GRU, as a variant of Recurrent Neural Network (RNN), has the ability to process time-series data and capture temporal dependencies in EEG signals. By combining attention mechanisms, GRU can more effectively extract complex features from EEG signals, including both time-domain and frequency-domain information.

## 3 Dataset and model

### 3.1 Dataset

The BCI competition IV-2a (BCI-2a) dataset is an important electroencephalogram (EEG) dataset widely used in research and competitions on brain computer interfaces (BCI). This dataset consists of EEG data from 9 subjects who were required to perform four different motor imagery tasks during the experiment, namely left hand, right hand, foot, and tongue motor imagery. These motor imagery tasks are based on the BCI paradigm of prompts, requiring participants to imagine themselves performing corresponding movements according to the arrow prompts on the screen.

Each participant conducted two sessions on different dates, with each session consisting of 6 runs and 48 trials. Each trial corresponds to a complete experiment, starting from the subject seeing the prompt and ending with completing the imagination task. Each exercise imagination experiment takes 4 s, with a sampling frequency of 250 Hz. The collected EEG data is band-pass filtered between 0.5-100 Hz. During this process, EEG data is recorded for subsequent analysis and processing. This dataset contains a total of 5,184 samples.

**Figure 1:**
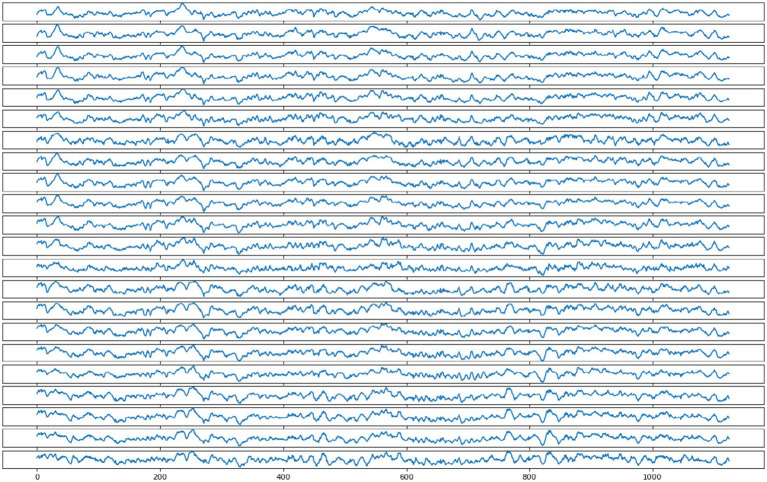
One of the EEG signal samples collected by subject 1.

Figure 1 shows one EEG signal sample collected by subject 1. The sample contains 22 channels, with each channel shown as a separate signal in Figure 1.

For the 9 subjects in the dataset, two sessions were conducted on different dates. Each session includes 288 samples generated by each participant. The samples from one session are used to train the model proposed in this paper, while the samples from the other session are used to evaluate the model.

### 3.2 Reinforcement learning attention model

**Figure 2:**
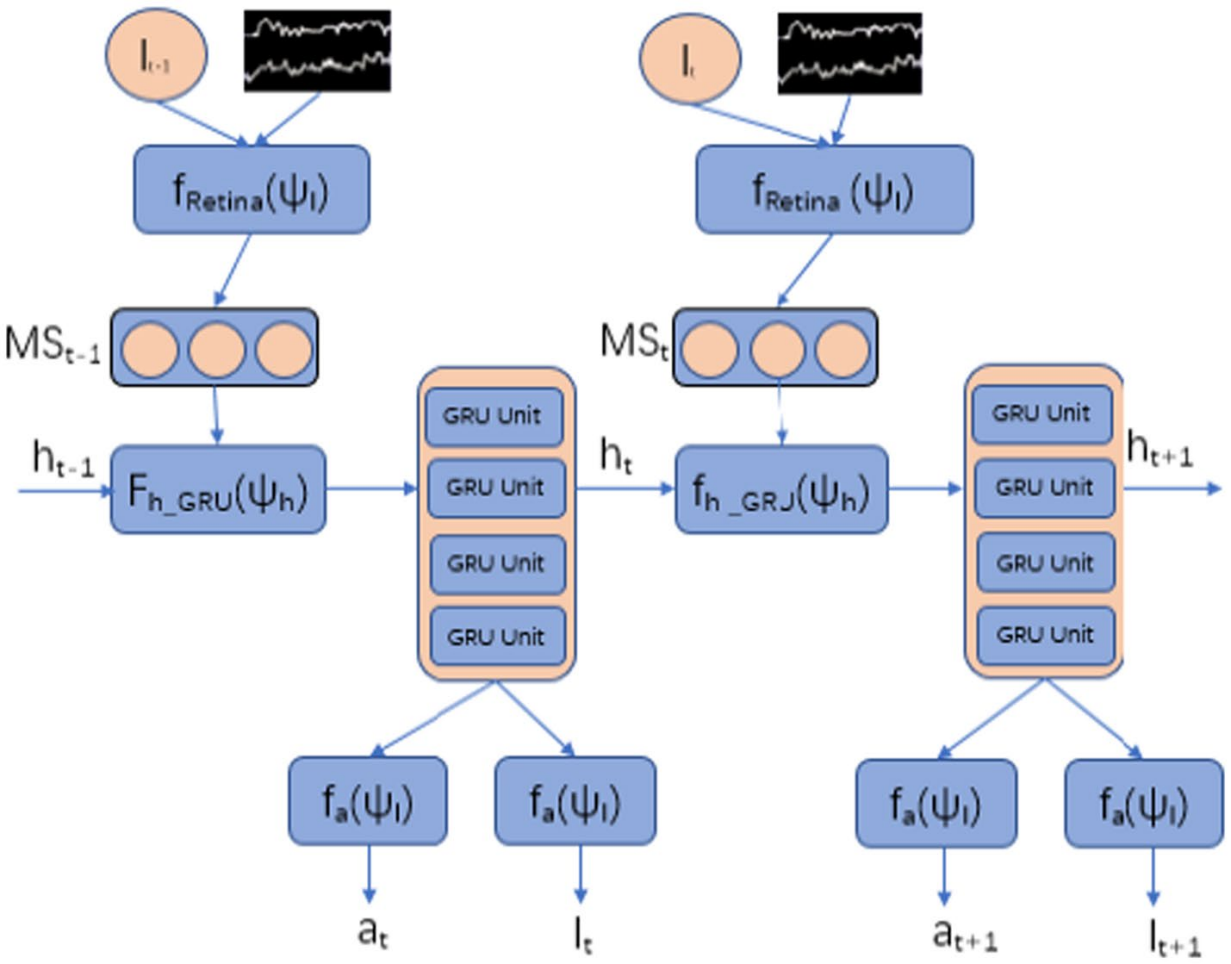
The architecture of reinforcement learning attention model.

Specifically, the agent is built using GRU as the basic neural network, as shown in Figure 2. It samples EEG signals through sensors in the time domain, gradually integrates global information over time, and takes action at the next time node through an action neural network.

#### 3.2.1 Multi scale receptors

At each time step t , the Agent observes the environment through EEG sampling sequences $x_{t}$ . Agent samples information from electroencephalogram (EEG) $x_{t}$ through limited field of view receptors $f_{\mathrm{Retina}}$ , observing several local EEG sequences.

Specifically, multi-scale sensors focus on multiple time-domain or frequency-domain domains related to tasks, and limited field of view receptors sample task related information near $l_{t-1}$ from EEG signals $x_{t}$ , which are formally represented as $f_{\mathrm{Retina}}\left(x_{t},l_{t-1}\right)$ . The selection of $l_{t-1}$ is achieved by the location network.

The multi-scale sensor is centered around $l_{t-1}$ and uses different resolutions from near to far. High resolution is used in the vicinity of $l_{t-1}$ , while resolution decreases sequentially in further positions. We refer to neural networks with different resolutions as multi-scale networks, which are implemented by sampling and integrating time-frequency domain EEG signals of varying lengths. A multi-scale network generates a multi-scale feature vector $MS_{t}$ through a multi-scale perceptron, as shown in Equation 1:


(1) $$MS_{t}=f_{MS}\left(x_{t},l_{t-1};\psi_{MS}\right)$$

Where $\psi_{MS}$ is the parameter of the multi-scale neural network.

#### 3.2.2 State sequence

At each time step, the Agent maintains a state sequence that is a mapping of environmental information (EEG signal sampling) collected by multi-scale sensors. That is, EEG signals are decoded into environmental knowledge and used as input for Agent action neural networks to generate actions, i.e., to determine where to deploy sensors. Thus, it can perceive specific EEG signal segments at specific locations and achieve an attention mechanism characterized by high selectivity. The state sequence of the Agent is generated by the hidden unit $h_{t}$ of the underlying network GRU, and each time step is dynamically updated, as shown in Equation 2:


(2) $$h_{t}=f_{h\_GRU}\left(h_{t-1},MS_{t};\psi_{h}\right)$$

Among them, the multi-scale sensor samples the feature vector $MS_{t}$ from the EEG signal as the output of the multi-scale neural network, completes the sampling of the EEG signal, and serves as the input of the GRU component.

#### 3.2.3 Action

At each time step, the action that the Agent needs to complete is implemented by a localization neural network, which determines the sampling position $l_{t}$ of the multi-scale sensor EEG signal through the localization neural network.

In the model proposed in this article, location actions are randomly selected from the parameterized distribution of location network $f_{l}\left(h_{t};\psi_{l}\right)$ at time t , which is obtained from Equation 3:


(3) $$l_{t}\sim p(\cdot |f_{l}\left(h_{t\_\mathrm{output}};\psi_{l}\right)$$

Here, $f_{l}$ represents the position neural network, and $\psi_{l}$ is the parameter of the neural network.

Similarly, environment actions are derived from a distribution conditional on the output of the action network, and the action at time t is obtained from Equation 4:


(4) $$a_{t}\sim p(\cdot |f_{a}\left(h_{t\_\mathrm{output}};\psi_{a}\right)$$

Here, $f_{a}$ represents the neural network that generates the action, $\psi_{a}$ is the parameter of the neural network, and $a_{t}$ is the action generated at time t .

For EEG signal classification, it is formulated using SOFTMAX output.

#### 3.2.4 Reward

The input of the intelligent agent is multi-scale sensors sampling EEG signals at specific positions $x^{t+1}$ and reward $r^{t+1}$ , with the goal of maximizing the cumulative reward R . The reward R is defined by Equation 5 as follows:


(5) $$R=\overset{T}{\underset{t=1}{\sum }}\gamma^{t-1}r^{t}$$

Here, the definition of reward signal $r^{t}$ is: $r^{t}=1$ if the EEG signal is correctly classified after t time steps, otherwise $r^{t}=0$ .

The model proposed in this article uses partially observable Markov decision processes, and the learning objective of the agent is a stochastic strategy P with parameter ψ , as shown in Equation 6:


(6) $$P=\pi \left(\left(l_{t},a_{t}\right)|s_{1:t};\psi \right)$$

Among them, the strategy function π is simulated by the GRU mentioned above, and $s_{1:t}$ is the mapping of the interaction between the agent and the environment, implemented by the hidden unit $h_{t}$ of the GRU, $s_{1:t}=x_{1},l_{1},a_{1},\ldots ,x_{t-1},l_{t-1},a_{t-1},\ldots ,x_{t},\ldots$ .

### 3.3 Training

Overall, the intelligent agent in the model proposed in this article is constrained by a set of parameters ψ , $\psi =\left\{\psi_{MS},\psi_{h},\psi_{a}\right\}$ , where $\psi_{MS}$ is the parameter of the multi-scale sensor, $\psi_{h}$ corresponds to the core network, and $\psi_{a}$ corresponds to the action network. By continuously optimizing this set of parameters to maximize cumulative rewards.

The above goal is essentially the continuous optimization of strategies by intelligent agents to achieve maximum returns, which is formally defined as Equation 7:


(7) $$J\left(\psi \right)=E_{p\left(s_{1:T};\psi \right)}\left[\overset{T}{\underset{t=1}{\sum }}r_{t}\right]=E_{p\left(s_{1:T};\psi \right)}\left[R\right]$$

$p\left(s_{1:T};\psi \right)$ is determined by strategy.

To accurately solve the above problem. It can be transformed into the following Equation 8:


(8) $$\begin{array}{l} \nabla_{\psi }J=\overset{T}{\underset{t=1}{\sum }}E_{p}\left(s_{1:T};\psi \right)\left[\nabla_{\psi }\log\pi \left(u_{t}|s_{1:t};\psi \right)R\right]\\ \approx \frac{1}{M}\overset{M}{\underset{i=1}{\sum }}\overset{T}{\underset{t=1}{\sum }}\nabla_{\psi }\log\pi \left(u_{t}^{i}|s_{1:t}^{i};\psi \right)R^{i} \end{array}$$

Among them, $s_{1:t}^{i}$ represents the state of the GRU hidden unit, which is the interaction result with environmental information, where i=1…M . $\nabla_{\psi }\log\pi \left(u_{t}^{i}|s_{1:t}^{i}|;\psi \right)$ is the gradient of GRU, which can be calculated using standard gradient backpropagation (Wierstra et al., 2007).

The working principle of learning rule is that the Agent samples the interaction sequence $s_{1:T}$ using the current strategy, and continuously optimizes the Agent parameter ψ to obtain a cumulative high reward, increasing the probability of corresponding actions that receive the cumulative high reward.

At the same time, there may be a high square difference between the above equation and. The state value function $E_{\pi }\left[R_{t}\right]$ can be introduced for optimization (Ingolfsson et al., 2020), as shown in Equation 9:


(9) $$\frac{1}{M}\overset{M}{\underset{i=1}{\sum }}\overset{T}{\underset{t=1}{\sum }}\nabla_{\psi }\log\pi \left(u_{t}^{i}|s_{1:t}^{i};\psi \right)\left(R_{t}^{i}-E_{\pi }\left[R_{t}\right]\right)$$

The value function plays a role in smoothing expected returns, utilizing historical information, guiding strategy selection, and balancing exploration and utilization in reinforcement learning, effectively avoiding high variance.

In addition, in our proposed model, the action network and multi-scale perceptron are trained through gradient descent method, while the localization network is trained through reinforcement learning.

## 4 Experiment

### 4.1 Method effectiveness verification

To validate the effectiveness of the algorithm, we evaluated our method on the toy dataset MNIST and translational MNIST. We first described the common design choices in all of our experiments:

Retina and position encoding: Retina encoding $f_{R\mathrm{etina}}\left(x,l\right)$ extracts k square plaques centered on position l . The size of the first plaque is $g_{w}\times g_{w}$ pixels, and the width of each adjacent plaque is twice that of the previous one. Then, adjust all k patches to $g_{w}\times g_{w}$ and assemble them together.
Multi scale perceptron network: Multi scale perceptron network $f_{MS}\left(x,l\right)$ consists of two ordinary fully connected layers. The output g of a multi-scale network is defined as $g=Rect\left(\mathrm{Linear}\left(h_{MS}\right)+\mathrm{Linear}\left(h_{l}\right)\right)$ , where $h_{MS}=Rect\left(\mathrm{Linear}\;\left(\rho \left(x,l\right)\right)\right)$ and $h_{l}=Rect\left(\mathrm{Linear}\;\left(l\right)\right)$ . For the model proposed in this article, the dimensions of $h_{g}$ and $h_{l}$ are 128, while the dimensions of g are 256.
  Location network: The strategy for location l is defined by a two component Gaussian with fixed variance. The position network outputs the average value of the position policy at time t and is defined as $f_{l}\left(h_{GRU}\right)=\mathrm{Linear}\left(h_{GRU}\right)$ , where $h_{GRU}$ is the state of GRU.
Core Network: In the model proposed in this article, the core network is Gated Recurrent Unit (GRU), which was used as the core network in experiments conducted on MNIST and EEG signal classification tasks.

**Figure 3:**
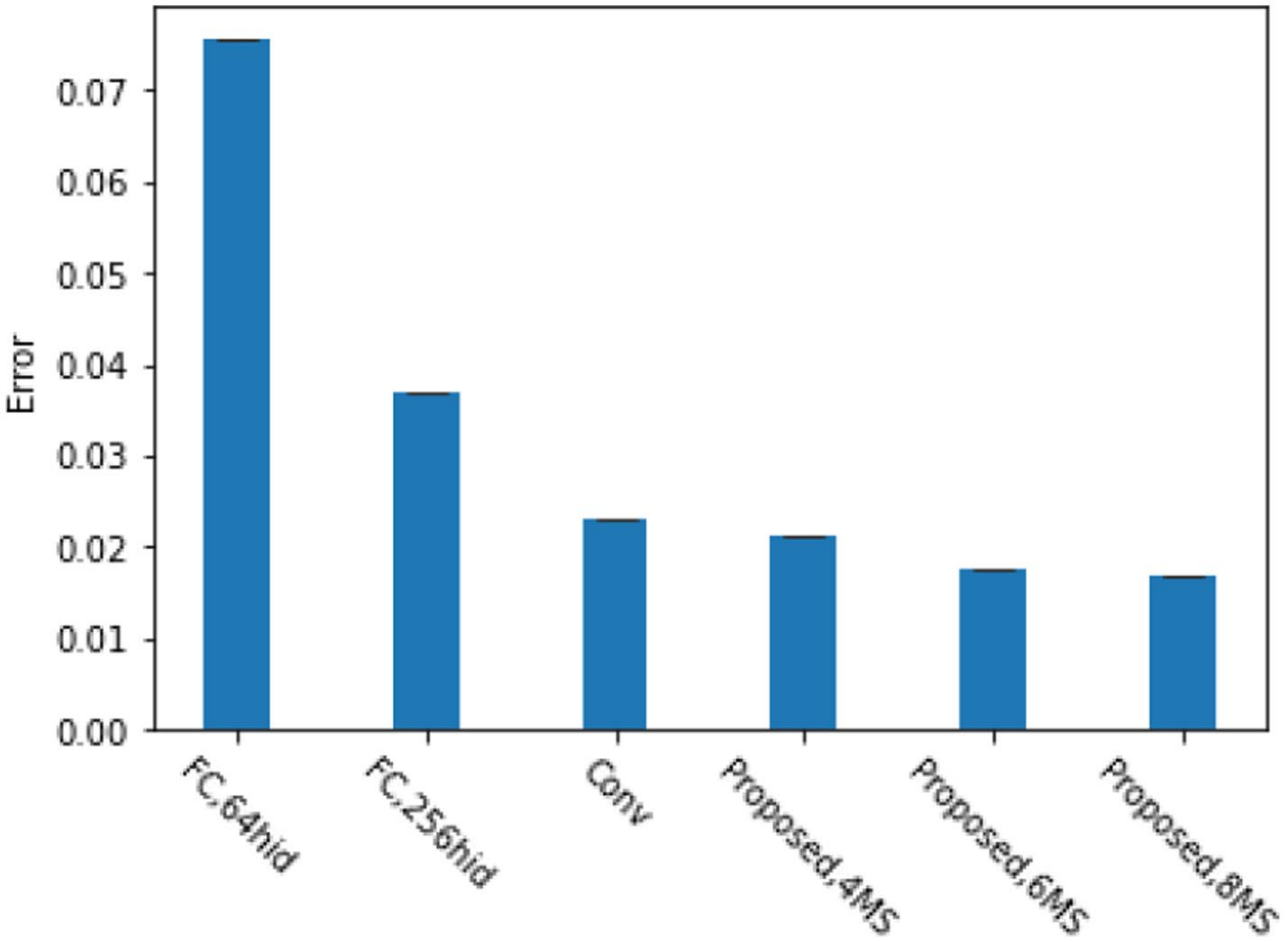
Comparison of various model errors under the translation of the MNIST dataset.

The translation MNIST dataset is a dataset generated based on MNIST, which is generated by placing numbers at a random position in a relatively larger blank image (such as 60×60 , 100×100 ), mainly to verify the feature capture ability of our proposed method. Figure 3 shows the classification error rate of the translated MNIST dataset with blank images of 60×60 . We chose two fully connected networks (64 and 256 units respectively) and a convolutional network (One convolutional layer, 8 10×10 convolutional kernels, fully connected layer with 256 units) for the comparison method. From the graph, it can be seen that when using four multi-scale perceptron, our proposed method achieves the same accuracy as convolutional neural networks. When using six multi-scale perceptron, our method achieves the best performance. This is because our proposed reinforcement learning based attention model can better focus attention on the object of interest. Meanwhile, experiments have shown that our proposed model can achieve good accuracy regardless of whether the numbers are centered or not.

### 4.2 Classification of EEG signals

Based on the BCI 2a dataset, this paper designed two different experiments to evaluate the proposed model. One is dependent on the evaluation of the subjects, and the other is independent evaluation with the subjects.

For subject dependent evaluation trials, the selection of training and testing sets is consistent with the original competition, where samples from one session are used for training and samples from another session are used for evaluation.

For subject independent evaluations, i.e., cross subject evaluations, we use samples from 8 subjects in the BCI 2a dataset as the training set, and samples from the remaining one subject as the testing set, such as samples from subjects 1–8 as the training set and samples from subjects 9 as the testing set.

In this experiment, the classification decision is only made at the $N^{th}$ time step, where the value of N is the dimension of a single sample. Action networks are implemented through fully connected networks. The number of hidden nodes in the basic network GRU is 256, and hyperparameters such as learning rate and positional policy variance are determined using random search. The total number of parameters for the entire model is 472,077. The determination and classification of rewards are directly related to whether they are correct. If the agent classification is correct, the reward for $N^{th}$ time step is 1, otherwise it is 0. In addition, the reward for all other time steps is 0.

We first tested the ability of our training method to successfully learn multi-scale perception strategies by using it to train a reinforcement learning based GRU model with up to 7 multi-scale perceptron networks on the BCI-2a EEG dataset. The multi-scale perceptron used in this experiment is just a 2×7 patch, which is only enough to capture a portion of two EEG signal channels. Therefore, the experiment also tested the ability of GRU based on reinforcement learning to combine information from multiple multi-scale networks. Please note that since the introduction of multi-scale networks is always random, a single multi-scale network is actually a classifier that can obtain a single random 2×7 patch as input. We also trained a standard feedforward neural network with a fully connected neural network layer of 256 neurons as the baseline. We see that each additional introduction of multi-scale networks improves the performance of GRU based on reinforcement learning until it reaches the minimum value of 6 multi-scale networks. Among the 6 multi-scale networks introduced, it has more advantages in classification performance on EEG datasets than models such as fully connected networks. This proves that the model can successfully learn to combine information from multiple multi-scale networks.

**Figure 4:**
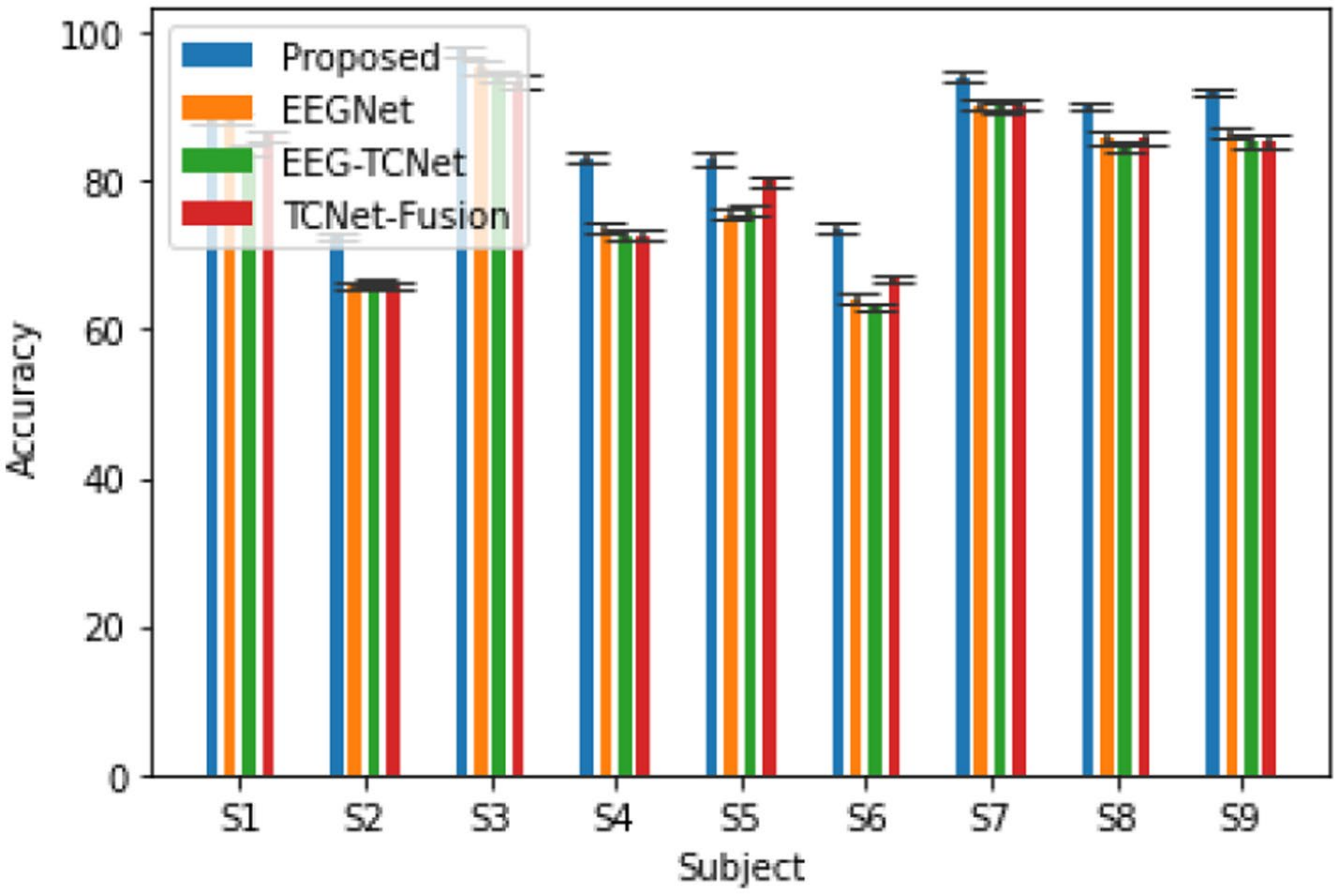
The accuracy of the proposed model and the comparison model in completing the subject dependent EEG signal 4-classification task on the BCI 2a dataset *κ*- fraction.

Figure 4 shows a comparison of the classification performance between the proposed model and some typical models in the field of motor imagery EEG signal classification (Altaheri et al., 2023), including EEGNet (Lawhern et al., 2018), EEG-TCNet (Ingolfsson et al., 2020), TCNet Fusion (Musallam et al., 2021), etc. The experiment used the same hyperparameter settings as the original text, and the preprocessing, training, and evaluation procedures were completely consistent. From the graph, it can be seen that the accuracy of the model proposed in this article is 86.78%, *κ*- The score is 0.83, achieving higher accuracy compared to the three comparison methods (Liu et al., 2023).

**Figure 5:**
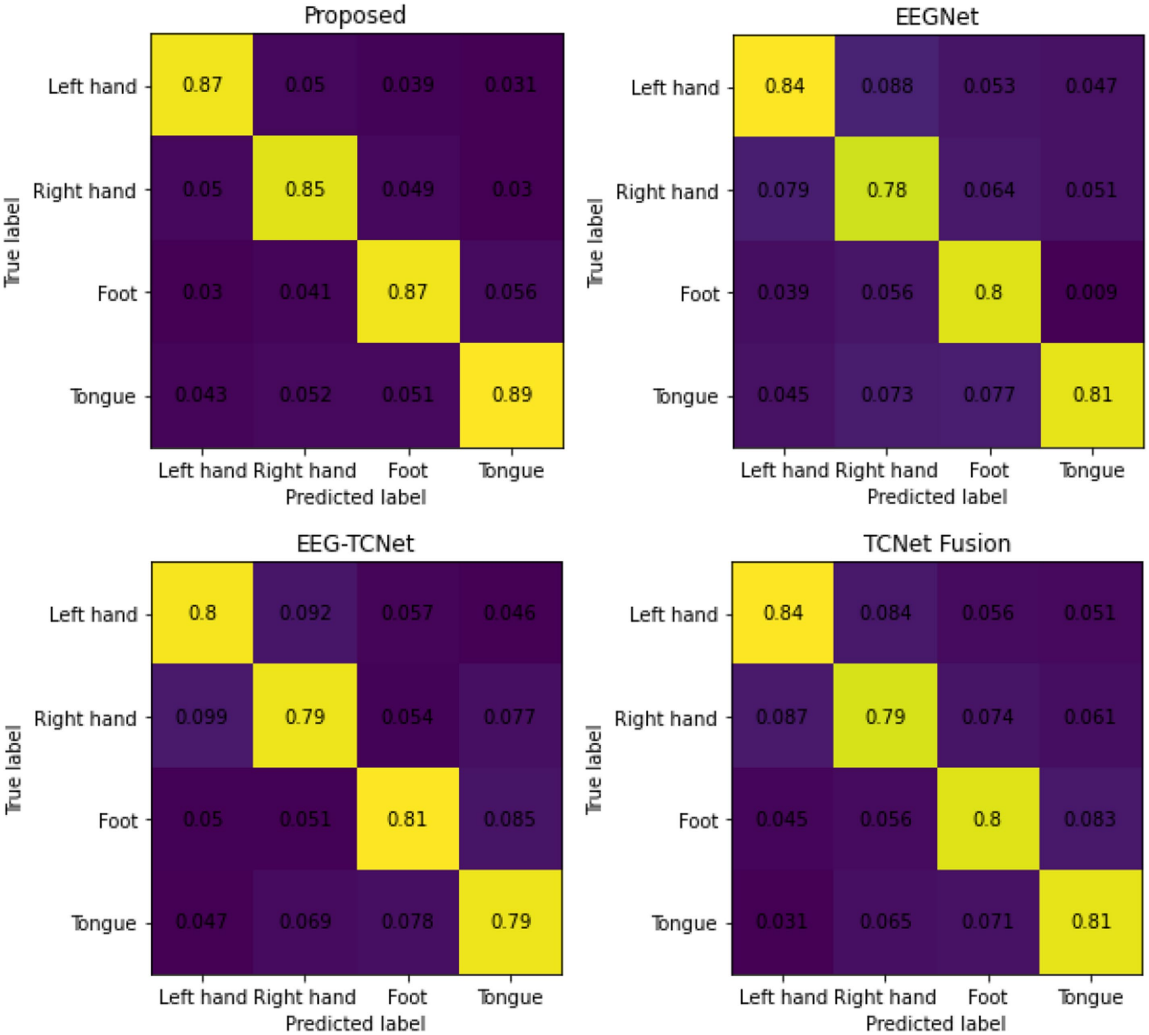
The average confusion matrix between the proposed model and the comparison model.

The average confusion matrix of the method proposed in this article and the three comparison methods is shown in Figure 5. As shown in the figure, the classification performance of the four types of motor imagery, including left hand, right hand, tongue, and foot, has achieved more ideal results.

**Figure 6:**
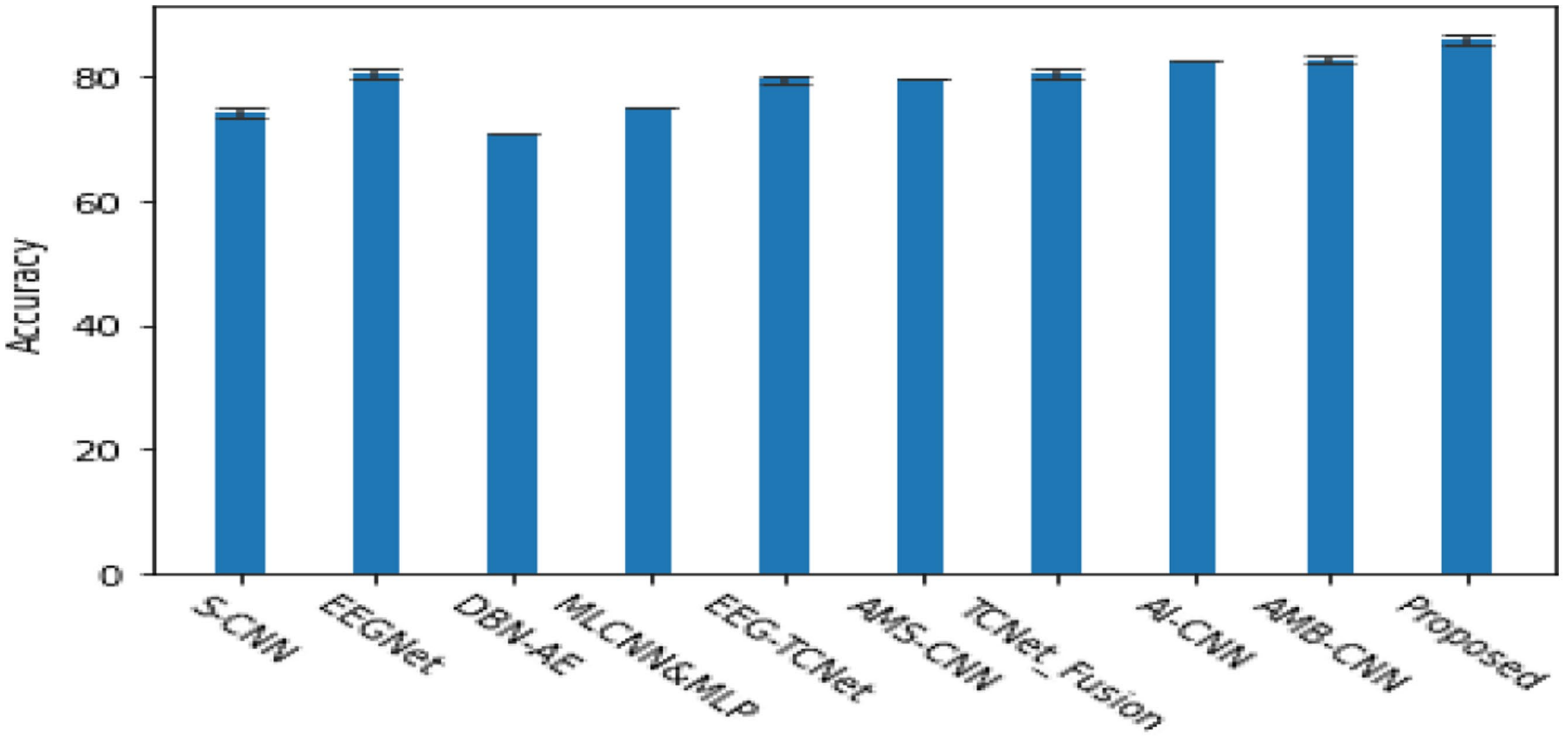
The cross validation accuracy of various classic algorithms in the subject 4-classification task, and κ- score.

Figure 6 shows a comparison of the classification accuracy between the proposed model and the most mature EEG classification algorithms currently available (Altaheri et al., 2023). The methods used in the comparison are S-CNN (Schirrmeister et al., 2017), EEGNet: CNN (Lawhern et al., 2018), DBN-AE (Hassanpour et al., 2019), MLCNN&MLP (Amin et al., 2019), EEG-TCNet (Ingolfsson et al., 2020), AMS-CNN (Li et al., 2020), TCNet-Fusion (Musallam et al., 2021), AI-CNN (Amin et al., 2022), AMB-CNN (Altuwaijri et al., 2022). The results show that the proposed algorithm can adaptively select EEG signal slices or position sequences using reinforcement learning methods and extract information from EEG signals only by processing the selected regions at high resolution. Compared with other state-of-the-art EEG signal classifications, it has shown certain advantages.

**Figure 7:**
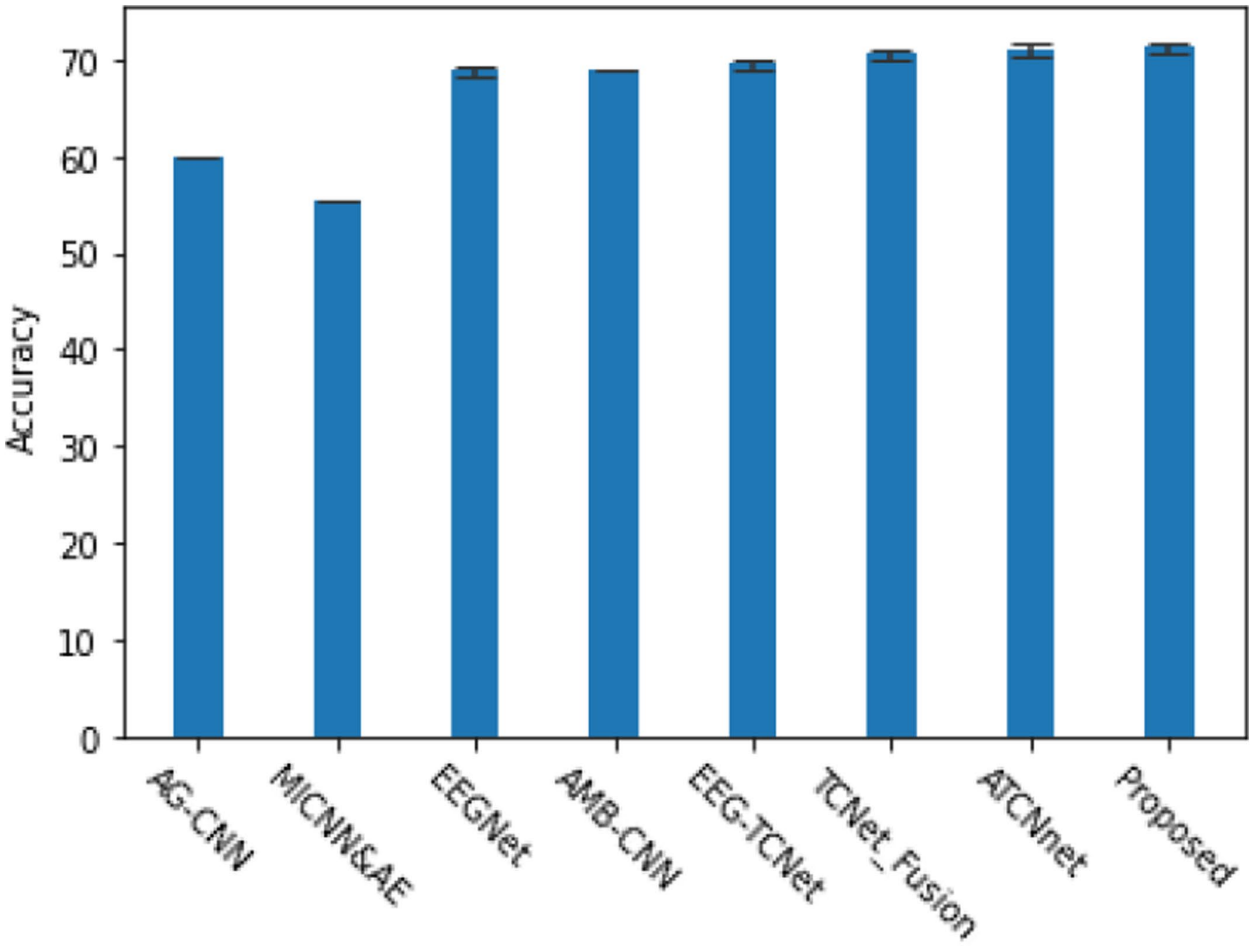
The cross validation accuracy and performance of various classic algorithms in subject independent (cross subject) 4-classification task and κ- score.

In addition to evaluating subject dependent EEG signal classification tasks, we also explored subject independent, i.e., cross subject EEG signal classification tasks, which are more important tests of model generalization ability. The methods used for comparison are AG-CNN (Zhang et al., 2020), MLCNN&AE (Amin et al., 2019), EEGNet (Lawhern et al., 2018), AMB-CNN (Altuwaijri et al., 2022), EEG-TCNet (Ingolfsson et al., 2020), TCNet-Fusion (Musallam et al., 2021), as shown in Figure 7. Based on the BCI 2a dataset, the proposed model demonstrated good generalization ability in cross validation experiments and had certain advantages in classification accuracy.

## 5 Conclusion

This article introduces a new attention model based on reinforcement learning GRU neural network. This network takes multi-scale windows as inputs, uses the internal state of GRU units to select the next focal position, and generates control signals in a dynamic environment. Although the model is non differentiable, the proposed unified architecture utilizes policy gradient methods for end-to-end training from input to action. This model has several attractive features. Firstly, in the field of EEG signal processing, attention models incorporating reinforcement learning principles can focus on key features, automatically filter out noise and redundant data, and enhance the accuracy of signal decoding. This mechanism effectively improves the robustness and adaptability of the model by continuously adjusting model parameters and flexibly responding to changing noise environments. Secondly, by leveraging deep interaction with dynamic environments, the attention mechanism based on reinforcement learning continuously optimizes the performance of the model, learning the characteristic information of each individual’s EEG signal. This personalized learning ability enhances the model’s tolerance for individual differences. At the same time, GRU can accurately capture the complex temporal dependencies in EEG signals and introduce attention mechanisms. GRU not only consolidates its analytical ability in the temporal dimension, but also greatly broadens the perspective of feature extraction, achieving efficient analysis of multidimensional information in both the time and frequency domains of EEG signals.

Experiments have shown that in nonlinear EEG signal classification tasks, GRU based on reinforcement learning can achieve competitive results compared to convolutional architectures and other machine learning based methods. In addition, our method allows for many interesting extensions. For example, another action can be used to enhance the network, allowing it to terminate at any point in time and make the final classification decision. This makes this method a potential EEG signal processing method that competes with models such as CNN and EEG net.

## Data Availability

Publicly available datasets were analyzed in this study. This data can be found here: https://www.bbci.de/competition/iv/#download.
